# Preparation and Performance of Transparent Thermal Insulation Coatings Based on Hollow Nano-TiO_2_ Spheres

**DOI:** 10.3390/ma19214448

**Published:** 2026-10-22

**Authors:** Pan Xie, Jinlong Fan, Xiaolin Zhang, Yong Jiang, Juan Du, Kexing Sheng, Sangang Lou, Yingjun Zhang

**Affiliations:** 1 School of Materials Science and Engineering, Sichuan University of Science and Engineering, Zigong 643000, China; 15982584810@163.com (P.X.);; 2 Material Corrosion and Protection Key Laboratory of Sichuan Province, Zigong 643000, China; 3 AVIC Chengfei Civil Aircraft Co., Ltd., Chengdu 610092, China; 4 Chengdu Hongrun Paint Co., Ltd., Chengdu 610000, China

**Keywords:** Nano-TiO_2_, transparent, thermal insulation, polyurethane

## Abstract

Hollow nano-TiO_2_ spheres (HNTSs) with shell coating ratios of 28.62%, 29.33%, 30.72%, 36.31%, and 38.17% were prepared by a sol–gel method. The novelty of this work lies in the use of the shell coating ratio as a tunable design parameter to balance visible-light transmittance and thermal insulation in waterborne polyurethane (WPU) coatings on glass. HNTS with a coating ratio of 30.72% provided the most balanced performance and was selected for composite optimization. The HNTSs were incorporated into WPU at loadings of 1, 2.5, and 5 wt%, and the resulting coatings were applied to glass substrates. At 5 wt% HNTS, the coating retained 72.19% visible-light transmittance and reduced the temperature rise rate to 1.94 °C/min, approximately 43% lower than that of blank glass (3.42 °C/min). These values compare favorably with previous reports of transparent TiO_2_-based and hollow-particle insulating coatings while requiring only a relatively low HNTS loading of 5 wt%. The low-refractive-index air encapsulated in the HNTS cavities lowers the effective refractive index of the composite, weakening visible-light scattering while maintaining transparency. In addition, the enclosed air cavities suppress solid-state heat conduction, and the TiO_2_ shells contribute to near-infrared scattering and attenuation. These combined effects demonstrate a practical design strategy for transparent thermal insulation in glass coatings.

## 1. Introduction

With the intensifying global energy crisis and rising environmental concerns, building energy efficiency has emerged as a critical priority for sustainable development. According to statistics, about 30% to 50% of heat loss in buildings occurs through windows and doors [1,2]. Therefore, developing materials that combine high transparency and excellent thermal insulation performance is of vital significance for reducing the energy consumption of building operations and achieving carbon neutrality targets [3].

Although traditional transparent insulation materials, such as low-emissivity glass, can effectively reduce radiative heat transfer by reflecting infrared radiation, they rely on complex preparation processes such as magnetron sputtering. This not only leads to high production costs but also makes the film prone to damage during processing, making it difficult to meet the demands of large-scale and low-cost applications [4,5]. In recent years, thermal insulation coatings based on nano-TiO_2_ and nano-SiO_2_ have shown great potential due to their cost-effectiveness and feasibility [6,7]. Among them, nano-TiO_2_ has attracted much attention due to its excellent photocatalytic performance [8,9], high chemical stability, and outstanding UV-shielding capability [10]. However, the inherently high refractive index of TiO_2_ causes substantial visible-light scattering and reflection, which reduces coating transmittance and makes it difficult to meet the transparency requirements [11]. This inherent trade-off limits its application in the field of transparent thermal insulation [12].

To overcome the inherent opacity of high-refractive-index thermal insulation coatings, researchers have turned to hollow microsphere architectures for optical property tuning. It has been established that hollow structures, which contain sealed air cavities (n ≈ 1.0) within their shells, can substantially lower the overall effective refractive index of the material [13]. For instance, Liu et al. [14] synthesized porous hollow SiO_2_ spheres with an ultra-low refractive index of 1.05, markedly lower than that of dense solid SiO_2_ nanospheres (typically 1.45–1.46 in the visible and near-IR regions). Furthermore, Lim et al. [15] demonstrated that when the average refractive index of hollow SiO_2_ nanospheres matches that of the polymer matrix, it enables the fabrication of optically transparent films that selectively reflect ultraviolet radiation.

Compared with SiO_2_, TiO_2_ possesses a significantly higher refractive index, which tends to induce pronounced visible-light scattering when incorporated directly into a resin matrix, thereby hindering the simultaneous attainment of high optical transmittance and adequate thermal insulation performance. The use of hollow structures has been recognized as a viable strategy to mitigate this trade-off. Typically, the sol–gel method has been utilized to coat polystyrene (PS) microsphere templates with a uniform TiO_2_ layer, yielding PS@TiO_2_ core–shell precursors; the subsequent calcination at 500 °C removes the organic template, producing hollow nano-TiO_2_ spheres (HNTSs) [15,16,17]. Bao et al. [12] demonstrated that the wall thickness and microstructure of the final hollow shells can be precisely tailored by adjusting the TiO_2_ coverage on the template surface. An appropriate shell coating ratio was found to ensure shell integrity, density, and uniformity, ultimately affording nanospheres with well-defined hollow interiors [18], low density, and reduced dielectric constants after calcination [19,20,21]. Despite these advances, the application of HNTS has been largely confined to photocatalysis and energy storage, while systematic investigations into their use in thermal insulation coatings remain notably scarce.

In this study, waterborne polyurethane resin (WPU) was selected as the polymer matrix due to its excellent film-forming ability and mechanical properties [22,23,24]. The HNTS/WPU composite coating was applied to glass substrates, and the influence of the TiO_2_ shell coating ratio on the hollow structure of HNTS, as well as on the visible-light transmittance and thermal insulation performance of the resulting coatings, was systematically investigated.

## 2. Experiment

### 2.1. Materials

Tetrabutyl titanate (TBOT, with a concentration of 98.0%), polystyrene (PS), and triethanolamine (with a concentration of ≥90.0%) were purchased from Zhichuan Technology Co., Ltd. (Jiangsu, China). Aqueous ammonia (analytical grade, concentration 25.0% to 28.0%) and anhydrous ethanol were supplied by Tianli Chemical Reagent Co., Ltd. (Tianjin, China) and Fuyu Fine Chemical Co., Ltd. (Tianjin, China), respectively. Waterborne polyurethane (LB-500) was obtained from Environmental Protection Rubber and Plastic Materials Co., Ltd. (Shenzhen, China). All chemical reagents were of analytical grade and used directly as received, without additional purification.

### 2.2. Preparation of HNTS

**Figure 1:**
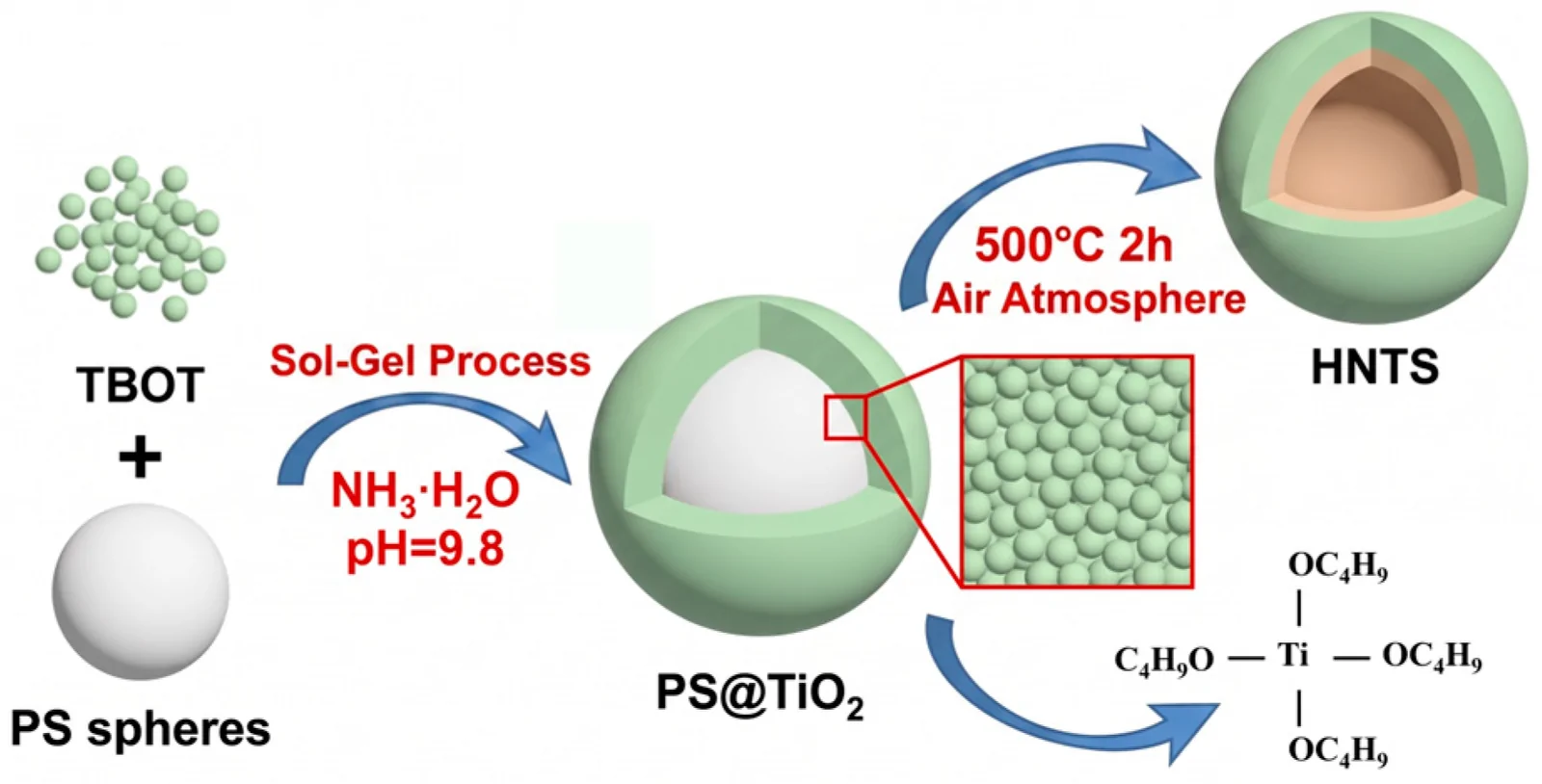
Schematic diagram of the HNTS preparation process.

PS@TiO_2_ core–shell spheres were synthesized via a sol–gel reaction using PS microspheres as the templates. In a typical procedure, 10 g of PS microspheres, 7.5 g of aqueous ammonia, and 80 mL of ethanol were placed into a three-necked flask under continuous stirring to form a uniform PS latex suspension. A precursor solution containing ethanol, TBOT, and triethanolamine was then added dropwise to the suspension with vigorous stirring. The resulting mixture was immediately transferred to an oil bath preheated to 80 °C and kept at this temperature for 6 h, followed by natural cooling to room temperature. The obtained products were collected by centrifugation, washed three times with ethanol, and dried at 60 °C for 12 h to yield PS@TiO_2_ spheres. Finally, the PS templates were removed by calcination at 500 °C for 2 h, producing hollow nano-TiO_2_ spheres (HNTSs). The entire fabrication process is schematically illustrated in Figure 1.

The precursor solution was prepared by dissolving 10 g of TBOT and 7.5 g of triethanolamine in 50 mL of anhydrous ethanol, corresponding to a TBOT/triethanolamine molar ratio of 1:1.7. The aqueous ammonia used was a 25–28 wt% ammonia solution.

### 2.3. Preparation of WPU/HNTS Coatings

The WPU/HNTS composite coatings were fabricated by a physical blending method. In a typical procedure, HNTS powder was dispersed in 15 mL of deionized water by ultrasonication for 10 min to obtain a homogeneous suspension. The suspension was subsequently mixed with the WPU and stirred at 80 °C for 4 h. Prior to coating deposition, glass substrates were ultrasonically cleaned in deionized water and ethanol sequentially to remove surface contaminants. The as-prepared WPU/HNTS mixture was then diluted with anhydrous ethanol and spray-coated onto the clean glass substrates at a loading of 10 mg/cm^2^. The spray-coating step was repeated three times, after which the specimens were dried at ambient temperature. The resultant coatings exhibited an average thickness of 16.8 ± 1.2 μm. Three sets of coatings were prepared with HNTS contents of 1 wt%, 2.5 wt%, and 5 wt% relative to the WPU matrix.

### 2.4. Analysis and Characterization

#### 2.4.1. Calculation of Coating Ratio

HNTSs with controllable coating ratios were prepared by controlling the diameter of the PS template, the sol–gel reaction temperature, and the initiator dosage. According to theoretical calculations, the relationship among the effective shell conversion rate, defined as the coating ratio (*i*), is related to the total mass of the calcined product (*m_f_*) and the initial mass of the PS template (*m_p_*) as follows:

*i* = (*m_f_*/*m_p_*) × 100% (1)

#### 2.4.2. Performance Characterization

The chemical structure of the PS@TiO_2_ coating was analyzed by Fourier transform infrared spectroscopy from PerkinElmer, Inc. (FTIR, Frontier, Shelton, CT, USA). The surface morphology and internal microstructure of HNTS and their corresponding composite coatings were investigated using field-emission scanning electron microscopy from Carl Zeiss (FE-SEM, Sigma300, Jena, Germany) and transmission electron microscopy from Hitachi High-Technologies (TEM, Hitachi HT7800, Tokyo, Japan), with energy-dispersive X-ray spectroscopy from Carl Zeiss (EDS, Sigma300, Germany) attached to the SEM for elemental analysis. The thermal conductivity and near-infrared (NIR) reflectance of the HNTS samples were determined using a thermal constant analyzer and an NIR reflectance spectrometer, respectively. The crystal structures of the samples before and after treatment were analyzed using X-ray diffraction from Bruker Corporation (XRD, D2 PHASER, Karlsruhe, Germany).

**Figure 2:**
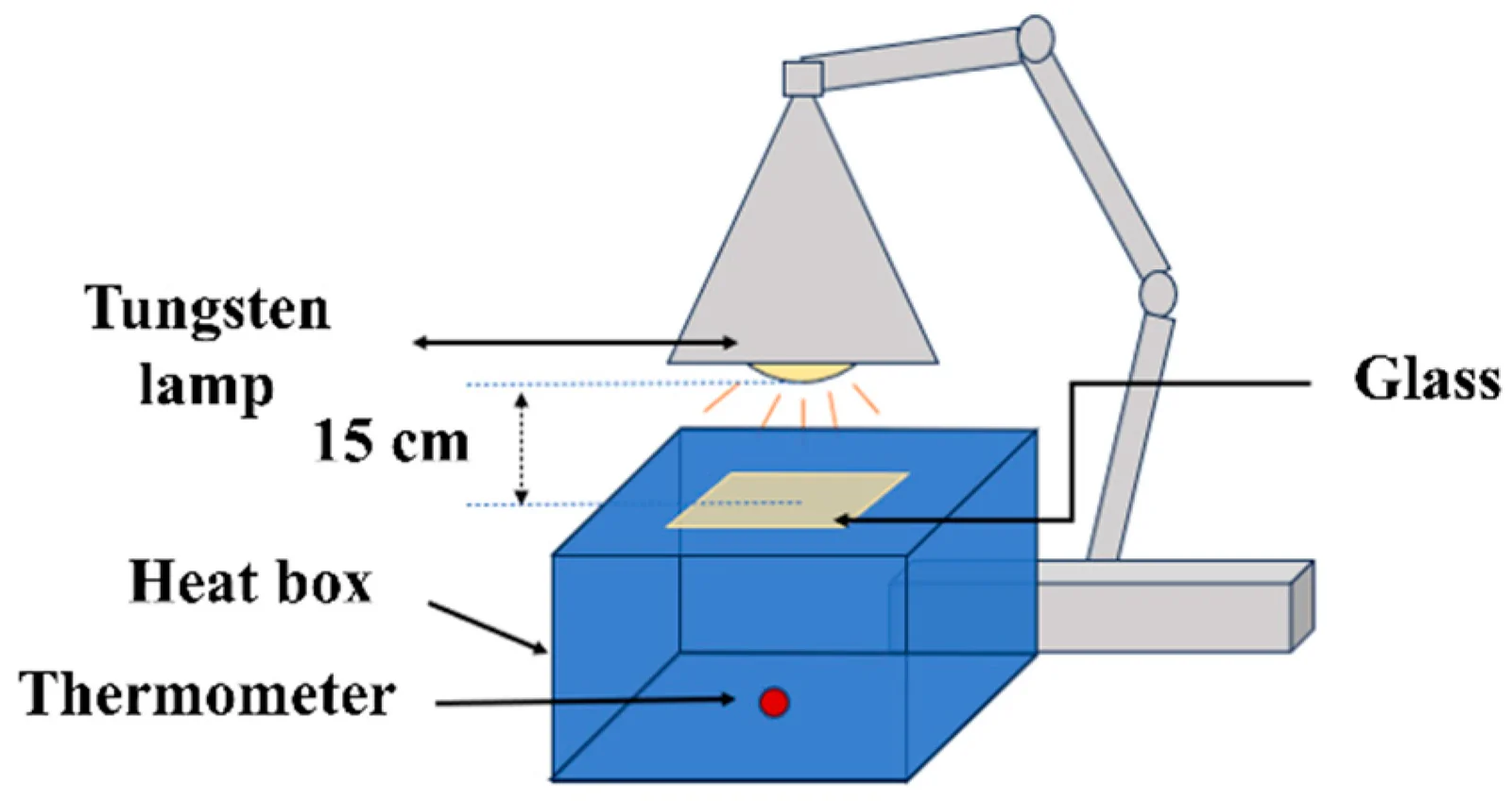
Simulated thermal insulation performance testing device.

The thermal insulation performance of the WPU/HNTS coatings was evaluated using a home-built setup based on the infrared reflection principle, as schematically shown in Figure 2. A closed polystyrene foam container was employed as a model room, with the window represented by the WPU/HNTS-coated glass. The insulating efficacy of the coated glass was tested by exposing it to a 100 W infrared lamp for 10 min, during which the temperature rise rate within the simulated enclosure was continuously recorded.

## 3. Results and Discussion

### 3.1. Formation Process of HNTS

**Figure 3:**
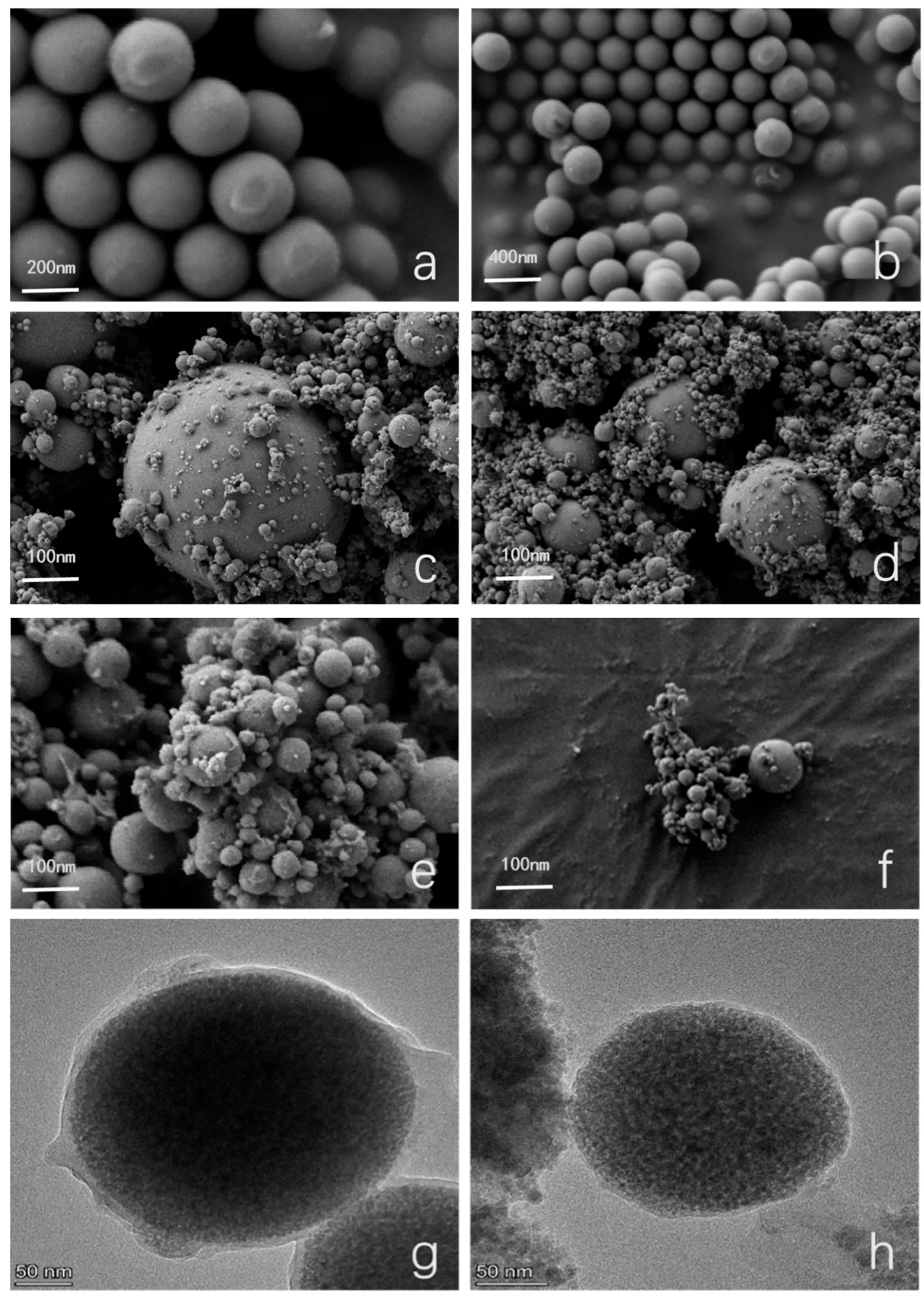

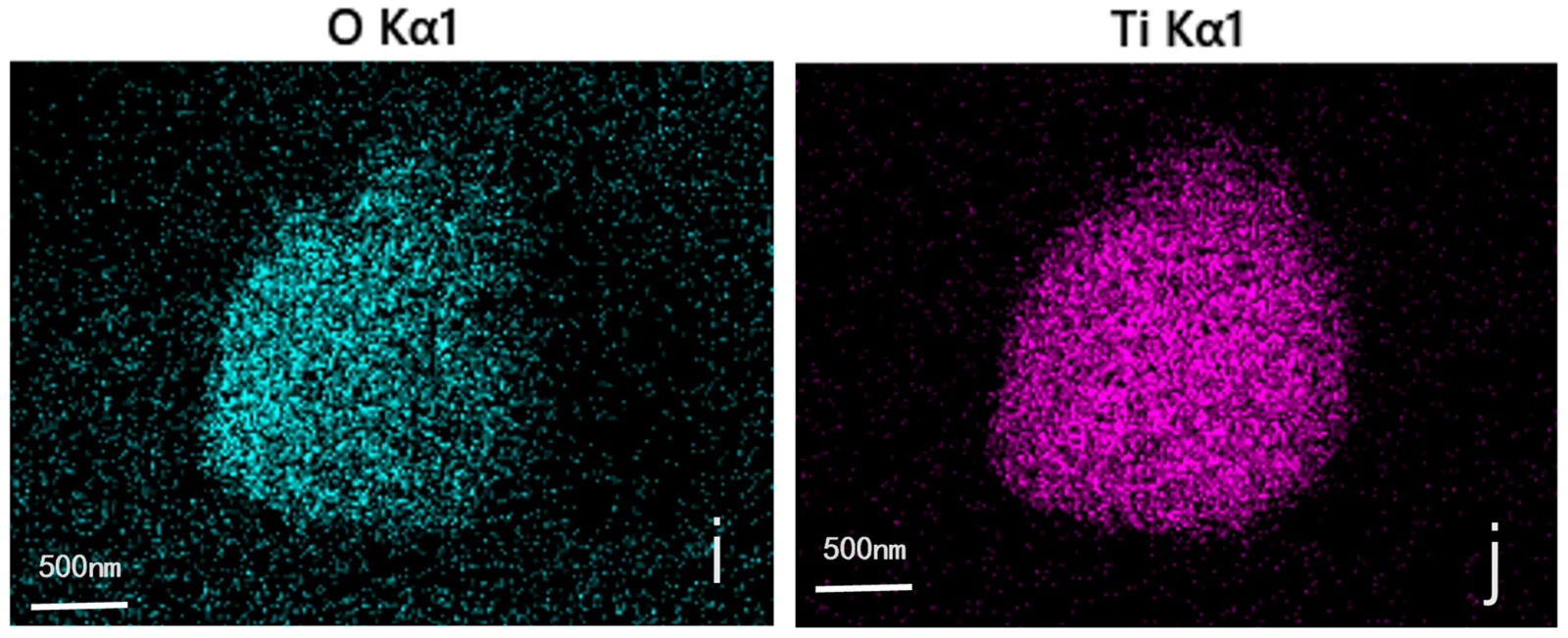
SEM images of (**a**,**b**) 200 nm PS particles; (**c**,**d**) PS@TiO_2_ spheres; (**e**,**f**) HNTS after calcination (**g**,**h**) TEM images of HNTS; (**i**,**j**) Corresponding EDS mapping of HNTS.

Figure 3 shows the characterization results of the microstructure and elemental distribution at different stages during the formation of HNTS. Based on the preparation process, the formation of HNTS can be divided into the following two steps.

The first step involves the formation of the PS@TiO_2_ core–shell structure. Figure 3a,b show that the selected monodisperse PS microsphere template has an average particle size of approximately 200 nm and a smooth surface. When TBOT underwent a hydrolysis–condensation reaction, TiO_2_ particles were formed in the colloidal solution. A TiO_2_ coating was then formed on the surface of the PS templates through the sol–gel reaction, resulting in a PS@TiO_2_ core–shell structure, as shown in Figure 3c,d. Zeta potential tests on the PS spheres revealed a Zeta potential of +11.4 mV, which facilitated the electrostatic adsorption of the TiO_2_ particles. Since the pH of the reaction system was 9.8, higher than the isoelectric point of TiO_2_, the Ti-OH groups on the TiO_2_ surface underwent deprotonation (Ti-O^−^), rendering the TiO_2_ particles negatively charged. These negatively charged TiO_2_ particles were consequently adsorbed onto the positively charged PS template surface via electrostatic attraction. The resulting PS@TiO_2_ spheres exhibited a rough surface, and the shell layer showed a distinct anatase phase, with TiO_2_ particle sizes ranging from 10 to 15 nm.

The second step is the removal of PS templates by calcination at 500 °C to yield HNTS. During this thermal treatment, the TiO_2_ shell underwent crystallization and densification, accompanied by slight shrinkage, which resulted in a reduced sphere diameter relative to that of the uncalcined PS@TiO_2_ precursors and an inward-contracted, well-defined hollow morphology (Figure 3e,f). This evolution arises from the concurrent removal of the organic PS core and the crystallization-induced reduction in the volume of the TiO_2_ shell. This process also introduced nanopores within the shell layer. The TEM images (Figure 3g,h) reveal the presence of fine pores, manifesting as inhomogeneous contrast features throughout the shell material. Elemental distribution maps (Figure 3i,j) demonstrate that Ti was homogeneously distributed across both the inner and outer surfaces of HNTS, supporting the successful formation of the hollow structure.

**Figure 4:**
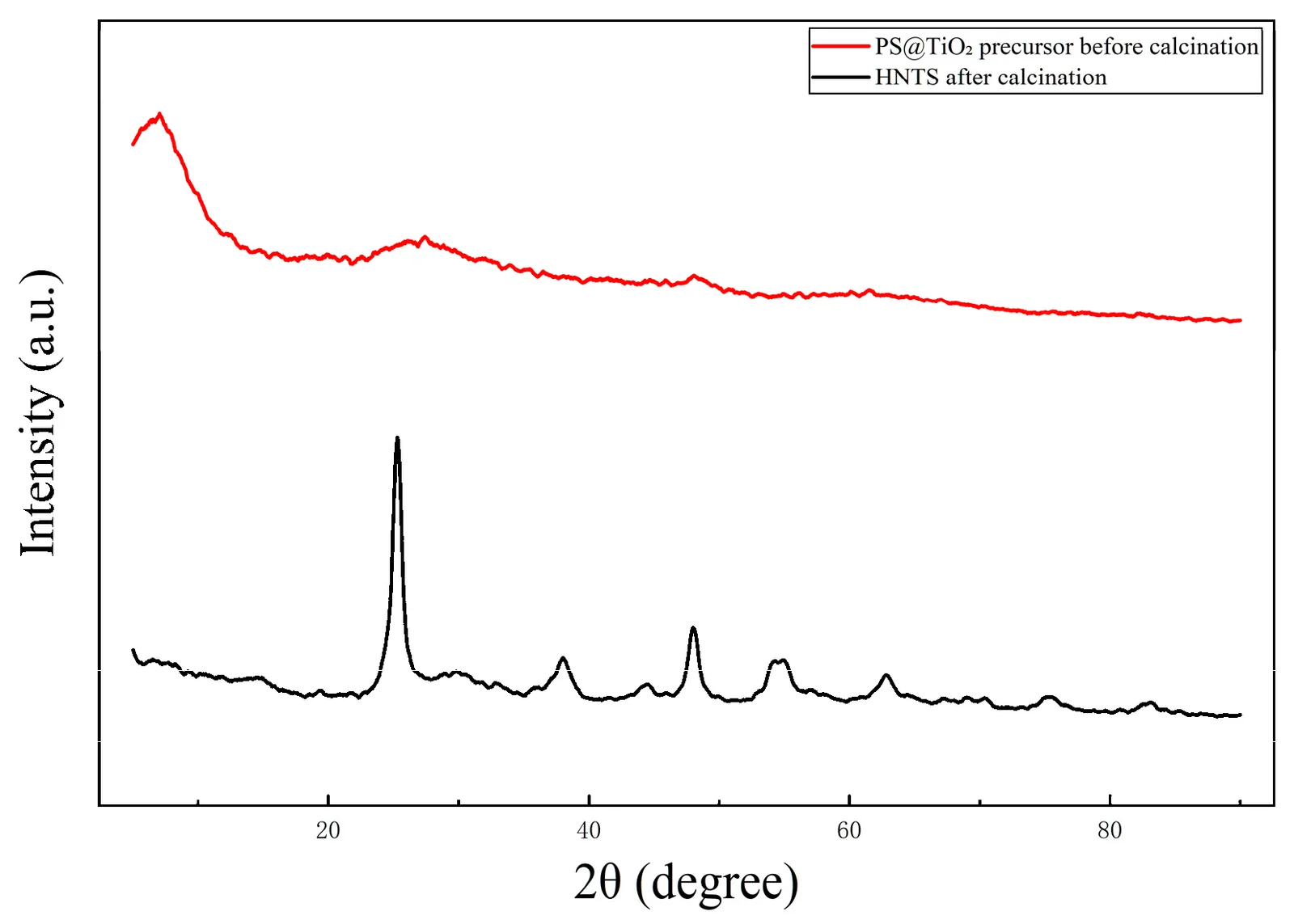
X-ray diffraction (XRD) patterns of the PS@TiO_2_ precursor before calcination (red line) and the HNTS obtained after calcination (black line).

The XRD patterns of the PS@TiO_2_ precursor before calcination and the HNTS obtained after calcination at 500 °C are compared in Figure 4. Before calcination, the pattern exhibits a broad low-angle scattering hump together with weak diffraction maxima, indicating low crystallinity and the presence of the PS template. After calcination, the low-angle hump disappears, while well-defined reflections appear at 2θ = 25.3°, 37.8°, 48.0°, 53.9°, 55.1°, and 62.7°. These reflections are indexed to the (101), (004), (200), (105), (211), and (204) planes of anatase TiO_2_ (JCPDS No. 21-1272). No distinct rutile (110) reflection is observed at 27.4°, indicating that no appreciable rutile phase is formed under the present calcination conditions. The stronger and sharper anatase peaks after calcination further confirm that removal of the PS template promotes crystallization and densification of the TiO_2_ shell while retaining the anatase phase.

### 3.2. Effect of HNTS Coating Ratio on Thermal Insulation Performance

To systematically study the influence of TiO_2_ coating ratio on the thermal insulation performance of WPU/HNTS coatings, HNTS with coating ratios of 28.62%, 29.33%, 30.72%, 36.31%, and 38.17% were prepared by regulating the sol–gel reaction process parameters. The HNTS loading in all samples was fixed at 5 wt% relative to WPU.

**Figure 5:**
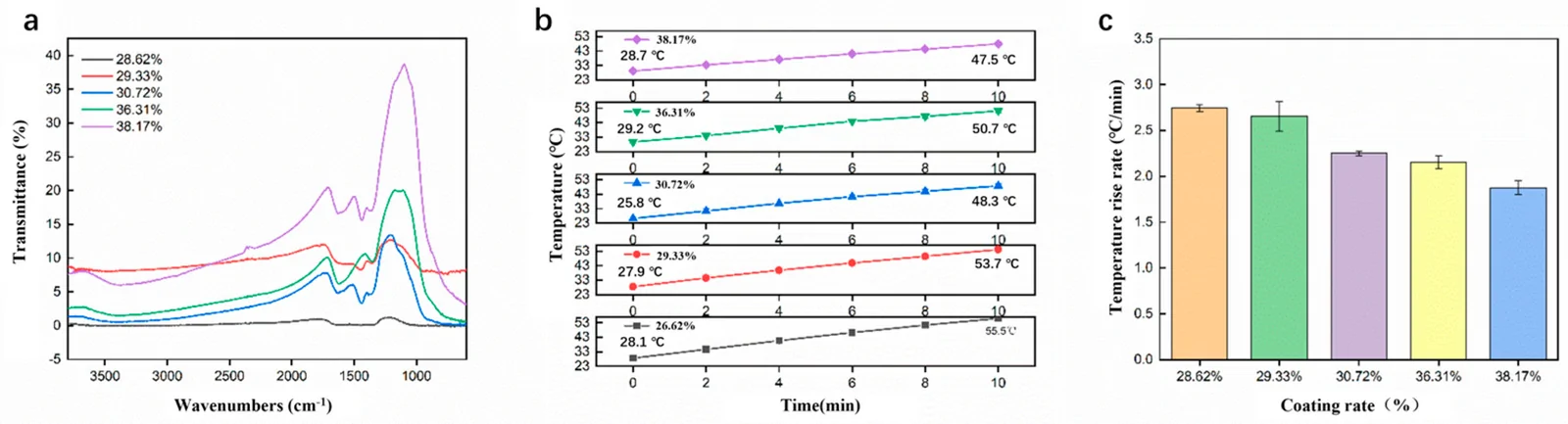
(**a**) FTIR transmittance spectra of HNTS with shell coating ratios of 28.62% (black line), 29.33% (red line), 30.72% (blue line), 36.31% (green line), and 38.17% (purple line); (**b**) temperature rise curves of the WPU/HNTS-coated glasses (5 wt% HNTS) with shell coating ratios of 28.62% (black squares), 29.33% (red circles), 30.72% (blue triangles), 36.31% (green inverted triangles), and 38.17% (purple diamonds). Error bars represent the standard deviation of three independent measurements; (**c**) Temperature rise rates of PS@TiO_2_ with different coating ratio Error bars represent the standard deviation of three independent measurements.

Figure 5a presents the FTIR transmittance spectra of HNTS with different shell coating ratios. As the shell coating ratio increases from 28.62% to 38.17%, the infrared transmittance decreases steadily, indicating enhanced attenuation of infrared radiation. This is attributed to the improved continuity and increased thickness of the TiO_2_ shell, which provide more sites for infrared scattering and absorption.

In addition to its NIR absorption role, TiO_2_ also functions as a thermal barrier filler owing to its reported low thermal conductivity of 0.14 W/(m·K) [25,26], which effectively suppresses heat conduction through solid-phase conduction, gas-phase conduction, and convection within the coating [27,28]. Furthermore, TiO_2_ acts as an infrared-reflective component, mitigating heat buildup and thereby reinforcing the overall thermal insulation performance [20].

To evaluate the thermal insulation performance of WPU/HNTS coatings, WPU/HNTS coatings with varying HNTS shell coating ratios were applied onto glass substrates and tested under a 100 W infrared lamp for 10 min using a home-built setup (Figure 2). Figure 5b presents the temperature rise curves recorded during continuous irradiation. Although the temperature of all coatings increases steadily over time, the heating rates differ markedly. The lowest heating rate of 1.89 °C/min is achieved at a coating ratio of 38.17%, indicating superior thermal insulation. Meanwhile, the coating with a coating ratio of 30.72% shows a slightly higher rate of 1.94 °C/min but exhibits the smallest fluctuations and the smoothest heating profile, indicating stable insulation behavior. These trends are further quantified in Figure 5c. At a coating ratio of 30.72%, the temperature rise rate is comparatively low with minimal variation, suggesting that the insulation performance is most stable under this condition—a benefit arising from the combined effects of the intact hollow structure and low effective thermal conductivity of the hollow HNTS [29,30,31,32,33]. Importantly, further increasing the coating ratio from 30.72% to 38.17% reduces the heating rate by only 0.05 °C/min, while requiring additional TiO_2_ and extended reaction time. Therefore, considering both cost-effectiveness and processing efficiency, 30.72% is identified as the optimal coating ratio.

### 3.3. Effect of HNTS Coating Ratio on Coating Transparency

**Figure 6:**
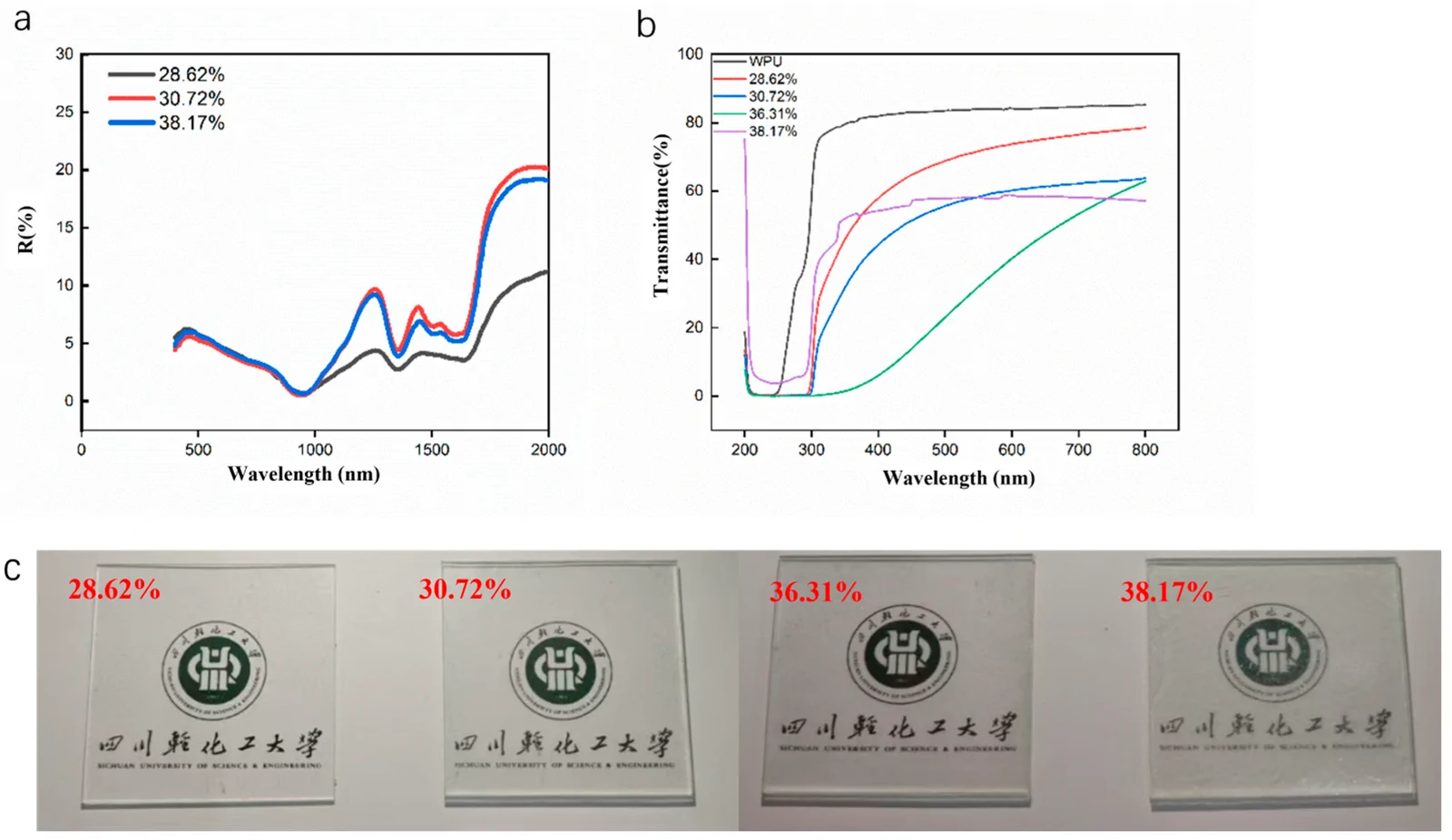
(**a**) Diffuse reflectance of HNTS with shell coating ratios of 28.62% (black line), 30.72% (red line), and 38.17% (blue line) in the range 400–2000 nm; (**b**) UV–Vis transmittance spectra of the bare WPU coating (black line) and the corresponding WPU/HNTS-coated glasses with shell coating ratios of 28.62% (red line), 30.72% (blue line), 36.31% (green line), and 38.17% (purple line) in the range 200–800 nm; (**c**) WPU/HNTS composite glass coatings with different coating rates, Digital photographs of the coatings on glass substrates for intuitive comparison of visible-light transmittance at 550 nm, which is adopted to characterize the visual transparency of the coatings.

The optical properties of the HNTS- and WPU/HNTS-coated glass were evaluated using UV-Vis diffuse reflectance spectroscopy (200–800 nm) and UV-Vis-NIR spectrophotometry (400–2000 nm), respectively [34]. As presented in Figure 6a,b, the HNTS samples exhibit a diffuse reflectance below 15% across the visible region. This low reflectance is attributed to the mesoporous shell architecture, which substantially decreases the effective refractive index of the material [35]. The resulting refractive index matching between the shell, the internal air cavities, and the surrounding medium significantly suppresses light scattering, thereby undermining the primary prerequisite for efficient light scattering—namely, a high refractive index and high reflectance [36,37,38].

As shown in Figure 6b, with increasing shell coating ratio from 28.62% to 38.17%, the transmittance of the coating at 550 nm gradually decreases from 75.3% to 70.8%. A higher shell coating ratio leads to a thicker and more complete TiO_2_ shell and a higher TiO_2_ content. However, the inherent high refractive index of TiO_2_ inevitably increases the scattering of visible light, resulting in a decrease in transparency. This result indicates that the selection of the shell coating ratio requires a trade-off between thermal insulation performance and transparency: a shell coating ratio of 38.17% offers the best thermal insulation but the lowest transparency, with a visible-light transmittance of only 70.8%. A shell coating ratio of 28.62% has the highest transparency but insufficient thermal insulation. A shell coating ratio of 30.72 achieves a good balance between the two, with a visible-light transmittance of 73.5% and a temperature rise rate of 1.94 °C/min.

It is well established that when the surface roughness features are smaller than the wavelength of incident light, the coating allows light to transmit through without appreciable scattering, thereby appearing transparent [39,40,41]. Consistent with this principle, Figure 6c demonstrates that the optical transparency of the WPU/HNTS-coated glass decreases progressively as the HNTS coating ratio increases.

It is well established that TiO_2_ spheres possess a relatively high refractive index. With increasing shell coating ratio, the hollow TiO_2_ shell becomes more complete and has a higher TiO_2_ content, which consequently reduces the optical transparency of the coating. Notably, the WPU/HNTS coating with a shell coating ratio of 38.17% delivers the best thermal insulation performance, whereas the coating with a shell coating ratio of 28.62% offers the highest transparency. This trade-off originates from the synergistic interplay between the structural features and the chemical composition of HNTS.

### 3.4. Effect of HNTS Loading on Thermal Insulation and Transmittance of WPU/HNTS Coatings

Based on the optimization results in Section 3.2 and Section 3.3, the shell coating ratio of HNTS was fixed at 30.72%, and the effect of HNTS loading on the overall coating performance was further examined.

**Figure 7:**
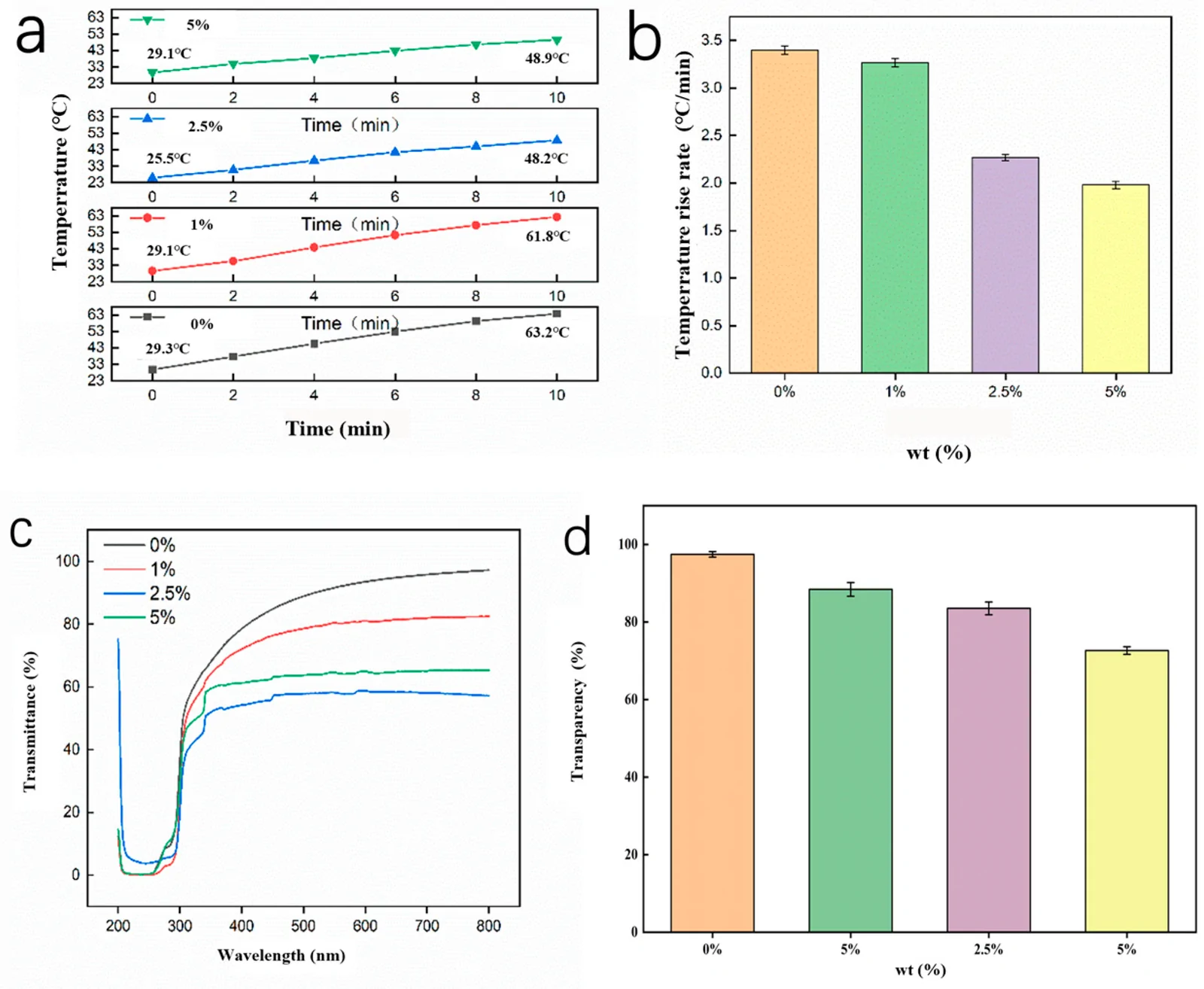

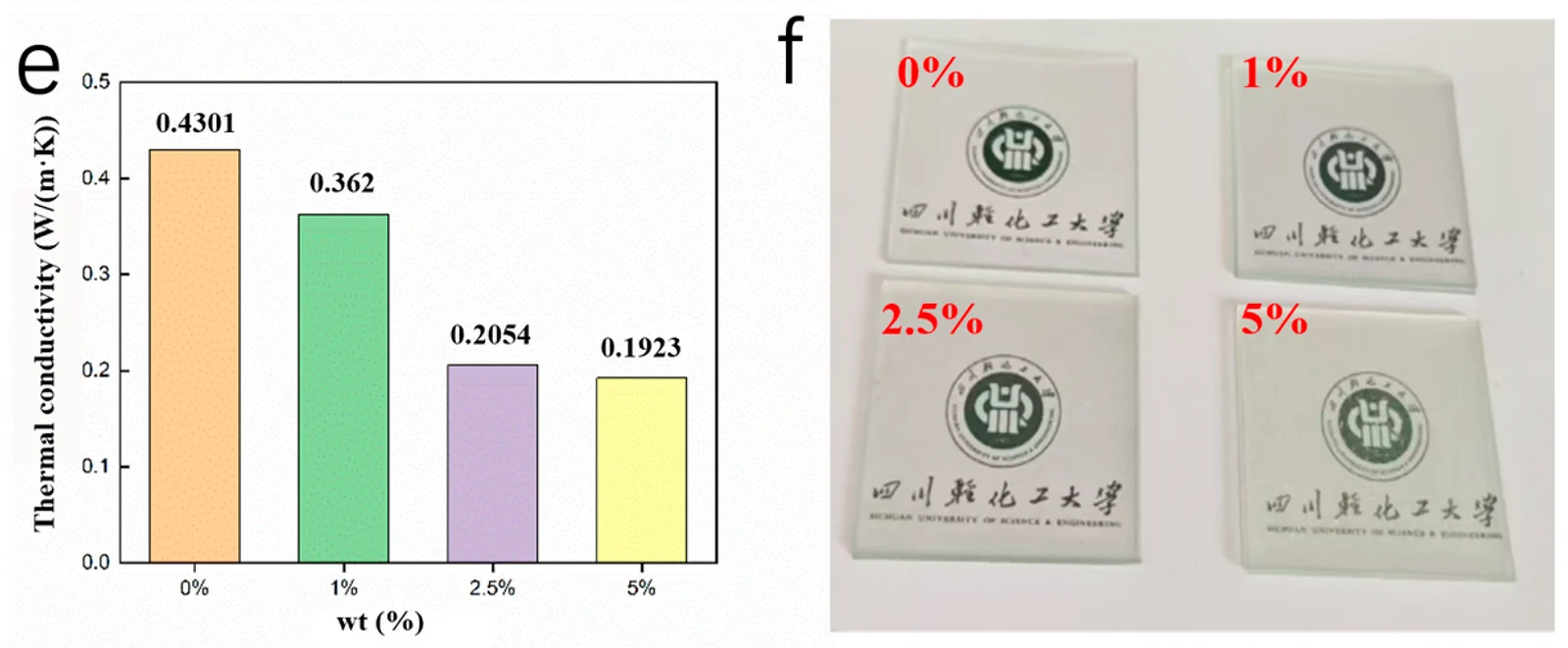
Effect of HNTS loading on WPU/HNTS coating performance: (**a**) temperature rise curves of the coated glasses with HNTS loadings of 0 wt% (black squares), 1 wt% (red circles), 2.5 wt% (blue triangles), and 5 wt% (green inverted triangles); (**b**) heating rates; (**c**) UV–Vis transmittance spectra of the coatings with HNTS loadings of 0 wt% (black line), 1 wt% (red line), 2.5 wt% (blue line), and 5 wt% (green line); (**d**) optical transparency; (**e**) thermal conductivity; (**f**) Digital photographs of the coatings on glass substrates for intuitive comparison of visible-light transmittance at 550 nm, defined as the spectral transmittance at 550 nm, the peak wavelength of human photopic vision, which is adopted to characterize the visual transparency of the coatings. Error bars represent the standard deviation of three independent measurements.

Figure 7 presents the performance of WPU/HNTS coatings with varying HNTS loadings. As shown in Figure 7a, increasing the HNTS loading from 0 to 5 wt% led to a marked improvement in thermal insulation performance. Specifically, the temperature rise rate of the pristine WPU coating was 3.41 °C/min, which decreased to 2.78 °C/min at 1 wt%, 2.35 °C/min at 2.5 wt%, and further to 1.94 °C/min at 5 wt% HNTS loading, representing a reduction of approximately 43% compared to the pristine WPU coating. This outstanding insulation effect originates from the unique hollow architecture of HNTS. On one hand, the enclosed air cavities within the hollow spheres possess ultra-low thermal conductivity, effectively interrupting solid-state heat conduction pathways [42,43,44]. On the other hand, the TiO_2_ shells exhibit strong near-infrared scattering and absorption, further reducing radiative heat transfer.

Figure 7e shows that the thermal conductivity of the composite coating decreases markedly with increasing HNTS loading. The pristine WPU coating exhibited a thermal conductivity of 0.312 W/(m·K), which dropped to 0.192 W/(m·K) upon incorporation of 5 wt% HNTS, corresponding to a reduction of 38.5%. This decrease originates from two combined effects: the uniform dispersion of hollow particles within the polymer matrix prolongs the heat conduction pathways, while the static air trapped inside the cavities, owing to its low thermal conductivity, further suppresses heat transfer.

Figure 7c,d illustrate the optical performance of the coatings. The incorporation of HNTS leads to a moderate decrease in visible-light transmittance. Notably, even at a high HNTS loading of 5 wt%, the coating retained a visible-light transmittance of 72.19% (Figure 7f), demonstrating high visual transparency. This behavior can be attributed to three factors: (1) the hollow structure of HNTS substantially lowers the effective refractive index, bringing it close to those of the polymer matrix and glass substrate, thereby minimizing interfacial scattering; (2) the HNTS diameter (~200 nm) is comparable to or smaller than the visible-light wavelength, and the low effective refractive index contrast keeps scattering weak; and (3) the moderate loading combined with good dispersibility maintains a smooth coating surface, which preserves high optical transparency.

To further evaluate the comprehensive performance of the as-prepared WPU/HNTS coating, a comparison with previously reported transparent thermal insulation coatings is summarized in Table 1. At the identical filler loading of 5 wt%, the HNTS composite coating exhibits a more significant suppression of temperature rise rate (43% reduction vs. blank glass) than the reported WPU/DHTSs system (26% reduction), accompanied by a minor decrease in visible transmittance. In contrast to the nano-TiO_2_/PPG coating that requires ~16.7 wt% TiO_2_ filler, our coating achieves comparable insulation behavior at a much lower loading of 5 wt%. ATO-based coatings are widely used for IR blocking, whereas the hollow TiO_2_ design in this work takes advantage of encapsulated air cavities to reduce solid heat conduction and scatter near-infrared light, offering a cost-effective alternative. Overall, the HNTS with an optimized shell coating ratio achieves a favorable trade-off between visible transparency and thermal insulation at low filler dosage.

### 3.5. Thermal Insulation Mechanism of WPU/HNTS Coatings

**Figure 8:**
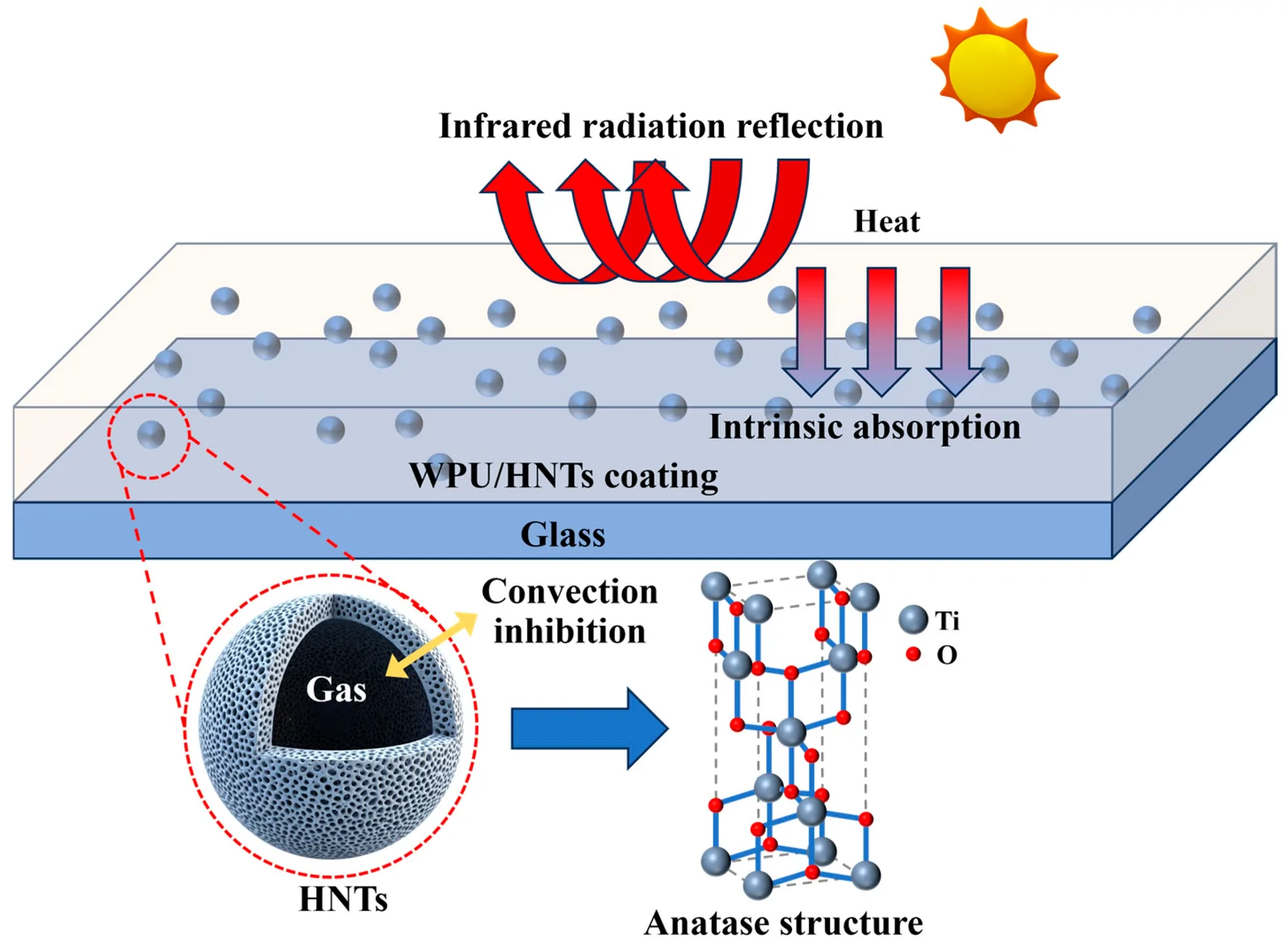
Schematic diagram of the thermal insulation mechanism of WPU.

Based on the results above, the transparent thermal insulation performance of the WPU/HNTS coating arises from multiple synergistic mechanisms, as schematically illustrated in Figure 8. At the optical level, anatase TiO_2_ possesses a band gap of ~3.2 eV, allowing it to absorb only UV radiation while exhibiting negligible intrinsic absorption in the visible spectrum, thereby helping to ensure high visible-light transmittance. Concurrently, the hollow architecture of HNTS lowers the effective refractive index of the TiO_2_ shell from its intrinsic value of ~2.5 down to 1.6–1.8, bringing it closer to those of the WPU binder and the glass substrate. This refractive index matching effectively suppresses scattering losses, which is key to preserving the optical transparency of the composite coating.

From a thermal insulation perspective, the internal cavities of HNTS encapsulate static air with a thermal conductivity of 0.026 W/(m·K), which effectively suppresses convective heat transfer within the confined space. The TiO_2_ shell itself has a reported thermal conductivity of 0.14 W/(m·K), and the hollow particles uniformly dispersed in the WPU matrix prolong the solid-state heat conduction pathways. Together, these effects markedly reduce the effective thermal conductivity of the composite coating.

From the perspective of thermal radiation management, the TiO_2_ shell intrinsically absorbs and scatters near-infrared radiation, converting a portion of the solar thermal energy within the coating itself. Meanwhile, the inherently low thermal conductivity of the composite effectively impedes the inward conduction of any absorbed heat, thereby providing a robust barrier against external thermal ingress.

The combined effects of these three mechanisms—optical transparency, thermal barrier effects, and radiative thermal management—enable the WPU/HNTS coating to preserve the essential daylighting function of architectural glazing while delivering outstanding thermal insulation performance.

## 4. Conclusions

In this study, HNTSs with tunable coating ratios were synthesized via a sol–gel method combined with calcination and subsequently compounded with WPU to fabricate high-transparency thermal-insulation glass coatings. The main conclusions are as follows:

(1) HNTSs with shell coating ratios ranging from 28.62% to 38.17% were successfully prepared by adjusting the sol–gel parameters. The resulting HNTS exhibit a regular hollow spherical morphology with TiO_2_ particle sizes of 10–15 nm. The internal cavities significantly reduce the effective refractive index of the HNTS particles and thus that of the composite coating.
(2) The shell coating ratio exerts a bidirectional influence: a higher shell coating ratio enhances near-infrared absorption and thermal insulation but reduces visible-light transmittance. A shell coating ratio of 30.72% was identified as optimal, offering balanced transparency and stable thermal insulation.
(3) At an HNTS loading of 5 wt% (with a shell coating ratio of 30.72%), the composite coating achieved optimal overall performance: a visible-light transmittance of 72.19%, a thermal conductivity of 0.1923 W/(m·K), and a temperature rise rate approximately 43% lower than that of uncoated blank glass.
(4) A combined mechanism involving three effects, consisting of refractive-index reduction for light transmission, hollow-cavity thermal blocking, and NIR absorption/scattering by the TiO_2_ shell, enables HNTS to overcome the inherent transparency–insulation trade-off of traditional TiO_2_-based coatings, providing a new design strategy for energy-saving building glazing.

## Figures and Tables

**Table 1:** Comparison of the present WPU/HNTS coating with representative reported transparent thermal-insulation systems.

System	Filler Content	Visible Transmittance (%)	Thermal Conductivity (W·m^−1^·K^−1^)	Reported Insulation Performance	Ref.
WPU/HNTS coating (this work)	5 wt%	72.19	0.1923	ΔT rise rate ~43% lower than uncoated glass	
WPU/DHTS composite coating	5 wt% (230 nm)	75	0.142	ΔT rise rate is 26% lower than uncoated glass	[3]
Nano-TiO_2_/PPG transparent coating	TiO_2_:PPG = 0.2:1 (~16.7 wt% TiO_2_)	75.0		UV reflectance 16.47%; NIR reflectance ~2.7–2.8%	[35]
ATO/nanosilica superhydrophobic coating		~70		Room temperature ~19% lower under identical irradiation	[23]
ATO/low-melting-glass transparent coating				IR transmittance (780–2500 nm) reduced by 13.3%; ΔT 4.7 °C lower than bare glass	[21]

## Data Availability

The data presented in this study are available on request from the corresponding author.
